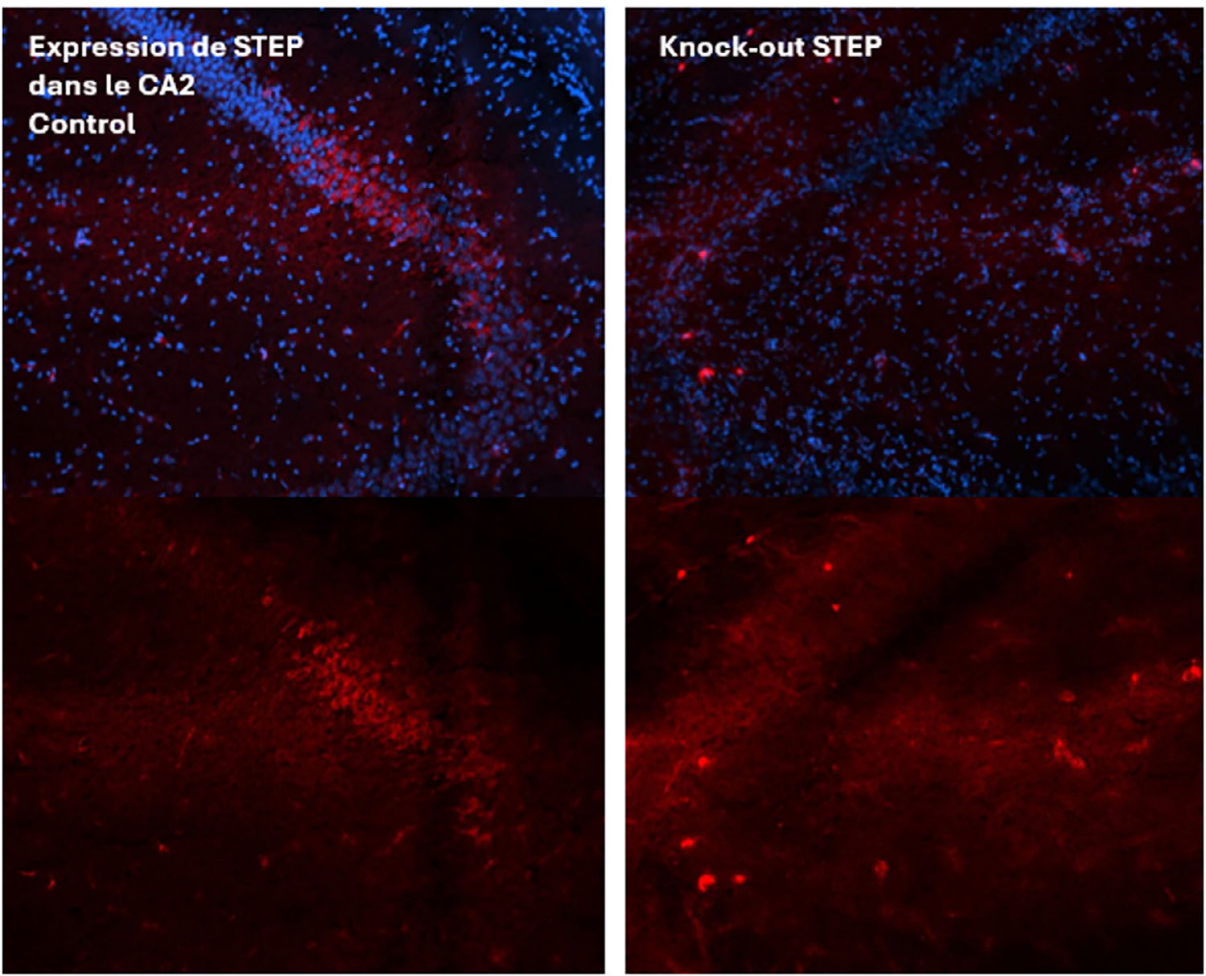# The effects of selective suppression of STEP in hippocampus region CA2 on social memory

**DOI:** 10.1002/alz70855_098482

**Published:** 2025-12-23

**Authors:** Laurie Bonenfant, Camilla Jane Alliance, Vincent Hervé, Klarissa Leduc, Daniel Lamontagne‐kam, Jonathan Brouillette

**Affiliations:** ^1^ Université de Montréal, Montréal, QC, Canada

## Abstract

**Background:**

Post‐mortem analyses of Alzheimer's patients reveal elevated levels of the STEP phosphatase (STriatal Enriched Phosphatase) in the brain. It is well‐established that STEP degradation activates cellular and molecular pathways critical for synaptogenesis and memory formation. While the CA2 region of the hippocampus is known to play a crucial role in social memory and exhibits high STEP expression, the specific role of STEP in this region remains unclear.

**Method:**

To investigate the role of STEP in the CA2, we are employing CRISPR‐Cas9 and Cre‐loxP technologies to selectively inhibit STEP expression in this region (Figure 1). We aim to determine whether localized STEP suppression affects social memory exclusively and whether its inhibition alters STEP substrates. Behavioral assessments include tests for social memory (social memory test, three‐chamber social interaction test, social interaction test, tube test), episodic memory (Morris water maze, object recognition test, passive avoidance test), anxiety (elevated plus maze), motor activity (open field test), and olfaction (odor discrimination test).

**Results:**

STEP deletion was successfully validated via immunofluorescence, and established protocols were used for all behavioral assays. We hypothesize that STEP deletion will impair social memory while leaving other forms of learning and memory unaffected.

**Conclusion:**

Identifying the STEP phosphatase as a key contributor to social memory in the CA2 region will enhance our understanding of the molecular mechanisms governing social behavior. This study will improve our understanding of social memory deficits associated with neuropathologies such as Alzheimer's Disease and can pave the way for the development of novel therapeutic strategies.